# The mechanism of tetraploidization in tree peony, and its implications for speciation and evolution of genus *Paeonia* L.

**DOI:** 10.3389/fpls.2025.1586225

**Published:** 2025-05-12

**Authors:** Yuan Zhong, Ming-jie Du, Run-ze Ji, Fang-yun Cheng

**Affiliations:** ^1^ State Key Laboratory of Efficient Production of Forest Resources, National Engineering Research Center for Floriculture, Peony International Institute, School of Landscape Architecture, Beijing Forestry University, Beijing, China; ^2^ Beijing Key Laboratory of Ornamental Plants Germplasm Innovation & Molecular Breeding, National Engineering Research Center for Floriculture, Peony International Institute, School of Landscape Architecture, Beijing Forestry University, Beijing, China

**Keywords:** karyotype, meiosis, GISH, FISH, hybrid, polypoid

## Abstract

**Introduction:**

Polyploidization is not only an important driving force for plant speciation and evolution, but also an effective approach for plant domestication and improvement. Polyploid taxa are quite common in *Paeonia* section *Paeonia* (herbaceous peonies), but very rare in section *Moutan* (tree peonies), which are known as the ‘king of flowers’.

**Methods:**

In this paper, we studied the origination of a tetraploid tree peony, *P. × lemoinei* ‘Golden Era’ (‘GE’, AABB), by comparing its karyotype with its seed parent, *P. × lemoinei* ‘Golden Isles’ (‘GI’, AB), based on genomic *in situ* hybridization (GISH) and rDNA fluorescence *in situ* hybridization (FISH). The meiosis behaviors of ‘GI’ were observed to make clear the cytogenetic process of unreduced (2*n*) gamete generation.

**Results:**

Four chromosomes with inter-genome translocations were identified in ‘GE’, two of which might be reciprocal translocations. The 2*n* female gametes *via* first division restitution (FDR) from ‘GI’ might play an important role in tetraploidization of ‘GE’.

**Disucssion:**

The distant hybridization between intersterile species from different subsections of section *Moutan* probably promotes the tetraploidization of tree peonies by facilitating 2*n* gamete production. The mechanism of tetraploidization in section *Moutan* is highly consistent with that in section *Paeonia*, but is inseparable with the assist from mankind. The divergence of life history between tree peonies and herbaceous peonies is speculated to contribute to the different level of polyploidization, and distinct tempo of speciation and evolution, between section *Moutan* and section *Paeonia* in genus *Paeonia*. These findings bring new insights to polyploid breeding, speciation and evolution in genus *Paeonia*.

## 1 Introduction

Polyploidization is one of the most important driving forces for plant speciation and evolution (Alix et al., 2017; De Storme and Mason, 2014; Jiao et al., 2011; Rieseberg and Willis, 2007; Soltis et al., 2009). The effects of polyploidy such as enlarged organs, vigorous growth and enhanced stress resistance are beneficial for plants to adapt to harsher environments in nature, and are also used to improve yield, quality, stress resistance and other important traits in plant breeding. So far, polyploid breeding has been widely carried out in the cultivation of new plant varieties in cereals, vegetables, fruits, trees, ornamental plants, and industrial crops (Chen, 2010; Fang and Morrell, 2016; Kang and Wei, 2022; Salman-Minkov et al., 2016; Zhang et al., 2019).

Tree peonies have been well known ornamental plants in China for more than 1000 years, and are popular in many other countries now (Cheng, 2007; Page, 2005). There are about 34 species in genus *Paeonia* (Paeoniaceae), which are classified into three sections (Cheng, 2007; Hong, 2010) or two subgenera (Hong, 2021). Most species of genus *Paeonia* are diploids (2*n*=2*x*=10), therein, only section *Paeonia* (belonging to subgenus *Paeonia* in the new taxonomic system of Hong (2021)) has natural tetraploid species, subspecies or populations (Hong, 2021). While all species of section *Moutan* (subgenus *Moutan* of Hong (2021)), known as tree peonies, are diploids, in addition, only two triploids (Zhong et al., 2024a; Li and Zhang, 1982) and several tetraploids (Zhong et al., 2024b; Zhong et al., 2023) have been found in the past decades in cultivated varieties. The tetraploid taxa of section *Paeonia* are mostly allotetraploids, which mainly originated from natural hybridization and polyploidization among diploid species. Moreover, there are some naturally formed autotetraploid populations in some species of section *Paeonia* (Ferguson and Sang, 2001; Sang et al., 2004; Zhou et al., 2021). Different from section *Paeonia*, the plants in section *Moutan* do not form polyploids under natural conditions (Hong, 2021), but only under artificial cultivation. There are great differences in the occurrence frequency and generation mode of polyploids between the two sections, implying that their polyploidization mechanisms might be different. Therefore, revealing the origination mechanism of polyploid tree peonies has important theoretical and practical significance for understanding the polyploidization mechanism of genus *Paeonia*, and promoting polyploid breeding of tree peonies.

The vast majority of the polyploid tree peonies are derived from *P. × lemoinei*, the artificially bred inter-subsectional hybrid tree peonies (Zhong et al., 2024a, 2024b, 2023). The inter-subsectional distant hybridization began around 1900s, with *P*. *delavayi* (B-genome) from subsection *Delavayanae* as the seed parents, and *P. suffruticosa* (A-genome) from subsection *Vaginatae* as the pollen parents (Hong, 2010; Page, 2005; Wister, 1995). Karyotype comparison based on GISH and rDNA FISH has shown that 2*n* gametes produced by the diploid seed parent, *P. × lemoinei* ‘High Noon’ (2*n*=2*x*=10, AB), play a key role in the formation of the allotriploid tree peony, *P. × lemoinei* ‘Oukan’ (2*n*=3*x*=15, ABC) (Zhong et al., 2024a), but the origination mechanism of allotetraploid tree peonies remains unclear. Although a few allotetraploid tree peonies have been found, the parents of them are mostly unknown. *P. × lemoinei* ‘Golden Era’ (‘GE’) is one of the earliest found allotetraploid tree peonies, which has higher fertility and better compatibility than many other cultivars in *P. × lemoinei* when crossing with herbaceous peonies, and is one of the most important pollen parents in intersectional distant hybridization (Page, 2005; Zhong et al., 2024b). The seed parent of ‘GE’ is *P. × lemoinei* ‘Golden Isles’ (‘GI’) (Page, 2005), a diploid intersubsectional hybrid tree peony. Although its pollen parent is unknown, ‘GE’ is still the best material available to study the origin of allotetraploid tree peony. The GISH study showed that the genome component of ‘GE’ is AABB (Zhong et al., 2024b), but its detailed karyotype information is lacking. Meanwhile, the karyotype of ‘GI’ has not been reported.

Unreduced gametogenesis is a key step in sexual polyploidization (Ramsey and Schemske, 1998). Different types of meiosis behaviors may produce unreduced (2*n*) gametes with diverse genetic compositions, thus affect the fertility and traits of polyploids (De Storme and Geelen, 2013; De Storme and Mason, 2014; Kreiner et al., 2017; Younis et al., 2014). Hence, the characteristics of meiosis behavior of diploid parents can provide important information to reveal the mechanism of polyploidization. Some studies have shown that ‘High Noon’ (2*n*=2*x*=10, AB) produces FDR (first division restitution) type 2*n* gametes through abnormal spindle localization, and transmits 10 chromosomes to the progeny intactly, resulting in the formation of the allotriploid, ‘Oukan’ (2*n*=3*x*=15, ABC) (Zhong et al., 2024a, 2019). However, the producing pathway and genetic type of 2*n* gametes from ‘GI’ are unclear.

Therefore, in this study, we used ‘GE’, a tetraploid tree peony, and its diploid seed parent, ‘GI’, as materials to conduct a karyotype comparison study by GISH and FISH, and observed the meiosis behaviors of ‘GI’. Based on the above results, the origination pathway of ‘GE’ was revealed, and the similarities and differences of polyploidization mechanisms between section *Moutan* and section *Paeonia* were discussed. The findings provide a theoretical basis for understanding the polyploidization mechanism of species in genus *Paeonia*, and for promoting polyploid breeding in section *Moutan*.

## 2 Materials and methods

### 2.1 Plant materials

Germinating buds of the tetraploid tree peony cultivar, *P.* × *lemoinei* ‘Golden Era’ (‘GE’), and its diploid seed parent, *P.* × *lemoinei* ‘Golden Isles’ (‘GI’), were collected for miotic chromosome preparations and karyotype analysis by GISH and FISH. Anthers of ‘GI’ were used for meiotic chromosome preparations and observation on meiosis behaviors. Fresh leaves of *P. delavayi*, one parent of *P.* × *lemoinei*, were employed for genomic DNA extraction and probe labeling for GISH in karyotype analysis of ‘GE’ and ‘GI’, and meiosis observation on ‘GI’, to distinguish the chromosomes of B-genome (*P. delavayi*), from those of A-genome (*P. suffruticosa*), the pollen parent of *P. × lemoinei.* All the materials were collected from living plants cultivated in the Peony Study Base of Beijing Forestry University, in the Jiufeng Forestry Park, Beijing.

### 2.2 Slides preparation

Germinating buds of ‘GE’ and ‘GI’ were taken in spring to prepare the metaphase chromosomes for karyotype analysis, according to the protocols described by Zhong et al. (2024a). Slides with well spread metaphase chromosomes were selected for the following *in situ* hybridization.

Young anthers of ‘GI’ in yellow green color were collected and fixed with Carnot fixative (anhydrous ethanol: glacial acetic acid = 3:1) at 4°C for 24 h, rinsed several times with distilled water, and then transferred to 70% ethanol, stored at -20°C in a refrigerator. Some of the fixed anthers were squashed on slides after rinsing with distilled water, and stained with modified Carbol-Fuchsin solution (G1165, Solarbio). The slides were observed under an optical microscope (Leica DM500, Germany) and photographed for meiosis observation.

Some other fixed anthers were processed as described by Dang et al. (2015) with some modifications for meiosis observation based on GISH. Pollen mother cells (PMCs) were suspended in mixed enzyme solution (3% cellulose + 0.3% pectinase + 1% snailase, W/V). Bottles were capped and vertically immersed in a 37°C water bath for 2.5 h. The suspension was then transferred into polyethylene centrifuge tubes and centrifuged at 2000 × g for 3 min. The supernatant was removed and the precipitate was resuspended in 200 μL Carnot fixative. Centrifugation was performed again as in the previous step. The precipitate was resuspended in 100 μL Carnot fixative. Then, the suspension was dropped on greaseless slides and dried rapidly using an alcohol flame.

### 2.3 Probe labeling and *in situ* hybridization

Genomic DNA extracted from young leaves of *P. delavayi* by plant genomic DNA kit (DP305, Tiangen) was used as GISH probe. 45S rDNA and 5S rDNA from maize were used as FISH probes. All the probes were labeled by nick translation kits, Dig-Nick Translation Mix (No. 11745816910, Roche) or Biotin-Nick Translation Mix (No. 11745824910, Roche), at 15°C for 90 min. The *in situ* hybridization and the following photographing were carried out according to the protocols described by Zhong et al. (2024a).

### 2.4 Karyotype analysis and meiosis observation

Karyotype analysis was performed based on the GISH and FISH signals integrated with relative length and arm ratio, according to the criteria summarized by Li and Chen (1985). For each plant material, 10 cells were selected for the measurement of karyotype data with Auto CAD 2019. Microsoft Office Excel 2016 and Adobe Photoshop 2020 were used for data processing and image postprocessing (Zhong et al., 2024a). The difference on arm ratios of homologous chromosomes between ‘GI’ and ‘GE’ were tested based on one-way ANOVA and LSD test using SPSS 18.0.

Meiosis behaviors were first observed and classified using the PMCs stained with Carbol-Fuchsin. Meanwhile, the behaviors of A- and B-genome sourced chromosomes in meiosis were observed using the PMCs marked with GISH. The number of monads, dyads, triads, and tetrads were counted with PMCs at the tetrad stage. The predicted frequency of 2*n* gametes were calculated using the following formula by Kondo et al. (2022): Frequency of 2*n* gamete (%) = (2×dyad + triad)/(monad + 2×dyad + 3×triad + 4×tetrad) × 100%.

## 3 Results

### 3.1 Karyotype of ‘GE’ and ‘GI’ revealed by GISH and FISH

**Figure 1:**
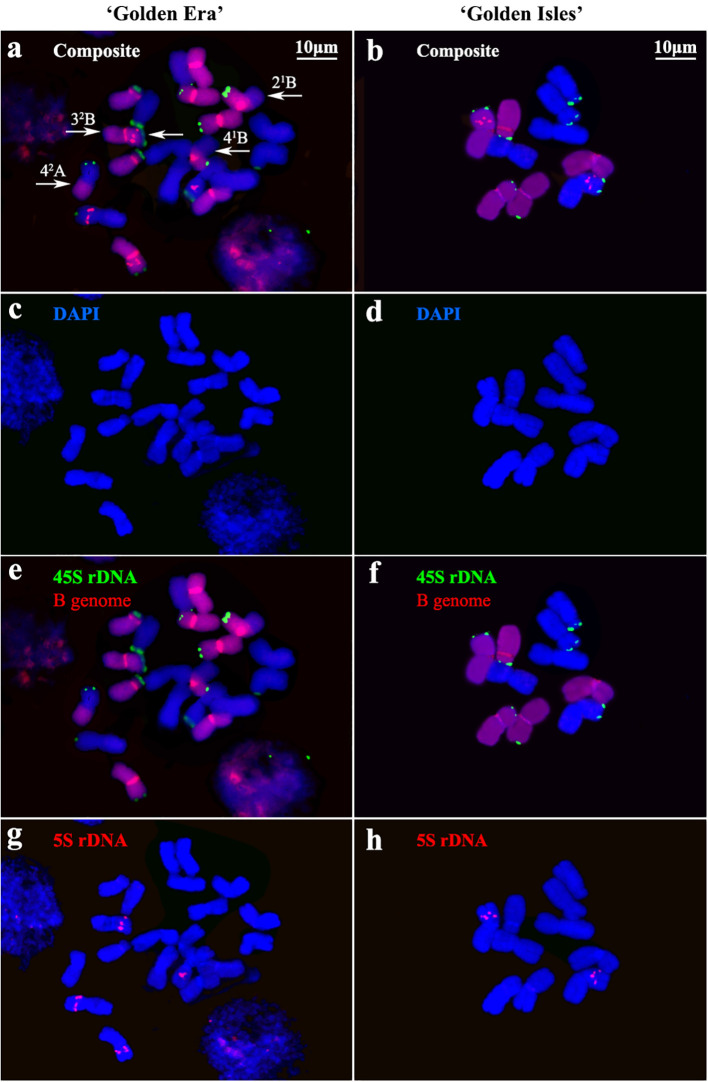
Chromosome identification based on genomic *in situ* hybridization (GISH) and fluorescence *in situ* hybridization (FISH) in ‘Golden Era’ and ‘Golden Isles’. **(a, b)**: Composite images with GISH and FISH signals for chromosome identification in ‘Golden Era’ and ‘Golden Isles’, white arrows show the inter-genome translocated fragments; **(c, d)**: DAPI stained chromosomes of ‘Golden Era’ and ‘Golden Isles’; **(e, f)** 45S rDNA sites (green) recognized by FISH and B genome (red) identified by GISH; **(g, h)**: 5S rDNA sites (red) recognized by FISH.

**Figure 2:**
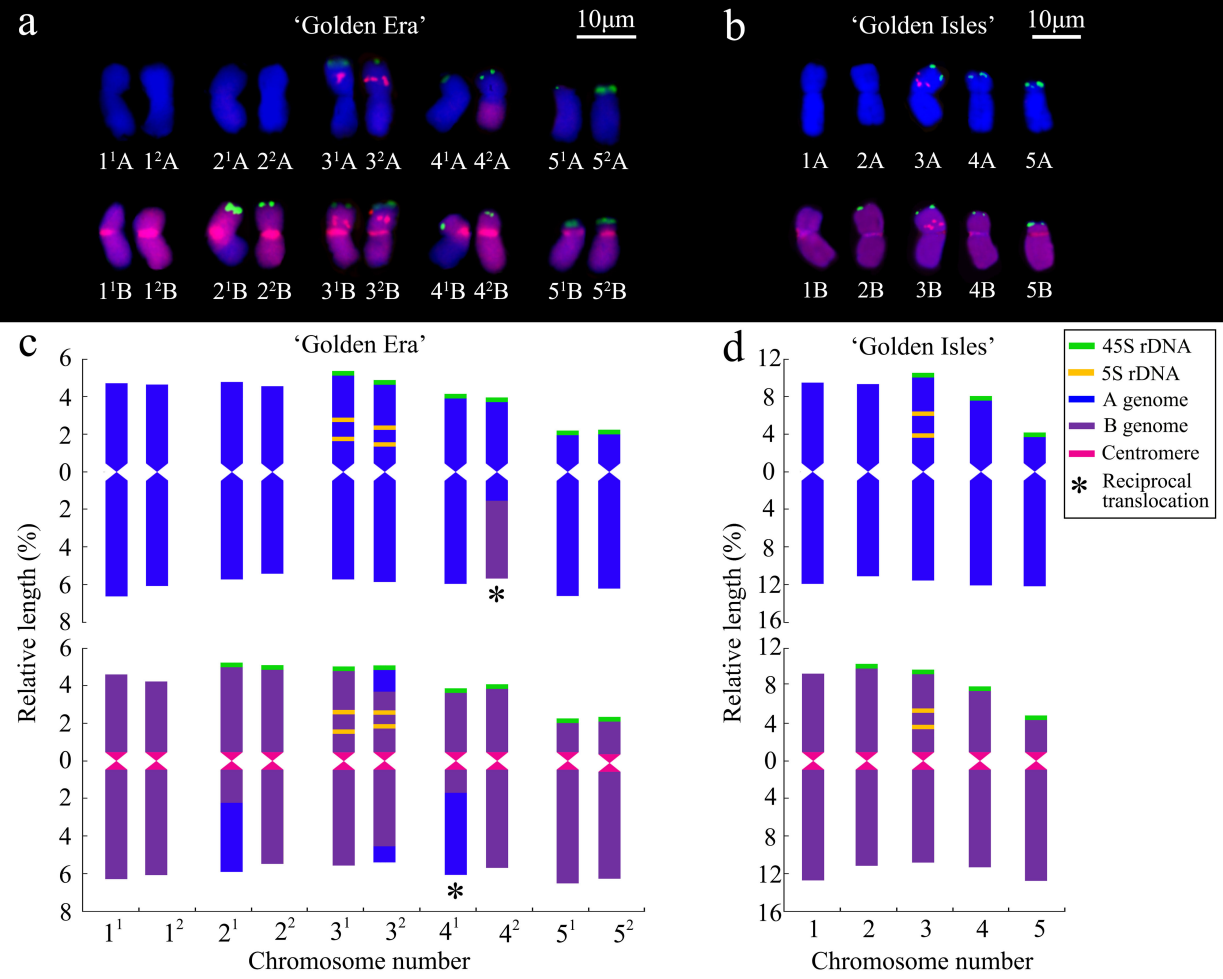
Karyograms **(a, b)** and ideograms **(c, d)** prepared with results from the genomic *in situ* hybridization (GISH) and fluorescence *in situ* hybridization (FISH) in ‘Golden Era’ **(a, c)** and ‘Golden Isles’ **(b, d)**.

The chromosomes of ‘GE’ and ‘GI’ were clearly distinguished into A- and B-genomes, according to the GISH signals with B-genome DNA as probes (**Figure 1**). FISH signals of 45S and 5S rDNA showed similar distribution patterns in ‘GE’ and ‘GI’. 45S rDNA signals are located on the end of short arm of 3A~5A and 2B~5B chromosomes. The 5S rDNA signals are located near the middle of the short arm of chromosome 3 of both A- and B-genomes (**Figures 1**, **2**).

Karyotype analysis based on GISH and FISH showed that ‘GE’ is an allotetraploid (2*n*=4*x*=20 = 15m+2sm+3st, AABB) (**Figure 2**; **Supplementary Table S1**). Chromosome 4 and 5 are heterozygous in arm ratio. The 4^1^A, 4^2^Aand 4^2^B chromosomes are all median (m) type, which are similar to chromosome 1~3, while 4^1^B chromosome is submedian (sm) type. Chromosome 5 of the two A-genomes and one of the B-genomes are subterminal (st) type, while only the 5^2^B chromosome is sm type. Among the 20 chromosomes of ‘GE’, 4 ones have inter-genome translocation segments, on the long arms of 4^2^A, 4^1^B and 2^1^B chromosomes, and on both long and short arms of the 3^2^B chromosome (**Figures 2a, c**; **Supplementary Table S1**). ‘GI’ is a diploid (2*n*=2*x*=10 = 8m+1sm+1st, AB), 5B chromosome of which is sm type, while 5A chromosome is st type. Both 1A~4A and 1B~4B chromosomes of ‘GI’ are m type (**Figures 2b, d**; **Supplementary Table S2**).

Although the 4^1^B chromosome in ‘GE’ was classified as sm type according to its arm ratio (1.71 ± 0.27), different from other 4B or 4A chromosomes in ‘GE’ (**Supplementary Table S1**). There was no significant difference on arm ratio between it and the 4B chromosome in ‘GI’ (**Supplementary Tables S2**, **S3**). The relative length of inter-genome translocation segments were 2.14 ± 0.28% and 2.15 ± 0.20% in 4^1^B and 4^2^A chromosomes (**Supplementary Table S1**), respectively. Therefore, the inter-genome translocations on the long arm of 4^1^B and 4^2^A chromosomes in ‘GE’ might be reciprocally translocated from the 4A and 4B chromosomes in ‘GI’, without changing their arm ratios significantly. The arm ratio of 5^2^B chromosome in ‘GE’ is similar to that of 5B chromosome in ‘GI’ (**Supplementary Tables S1**, **S2**), which is significantly smaller than that of 5^1^B chromosome in ‘GE’ (**Supplementary Table S3**), implying that the 5^2^B chromosome might come from the 5B chromosome in ‘GI’. In addition, the other seven chromosomes of ‘GI’ are also highly similar to their homologous chromosomes in ‘GE’, suggesting that 10 of ‘GE’’s 20 chromosomes are likely come from its seed parent, ‘GI’.

### 3.2 The meiosis behaviors of ‘GI’

**Figure 3:**
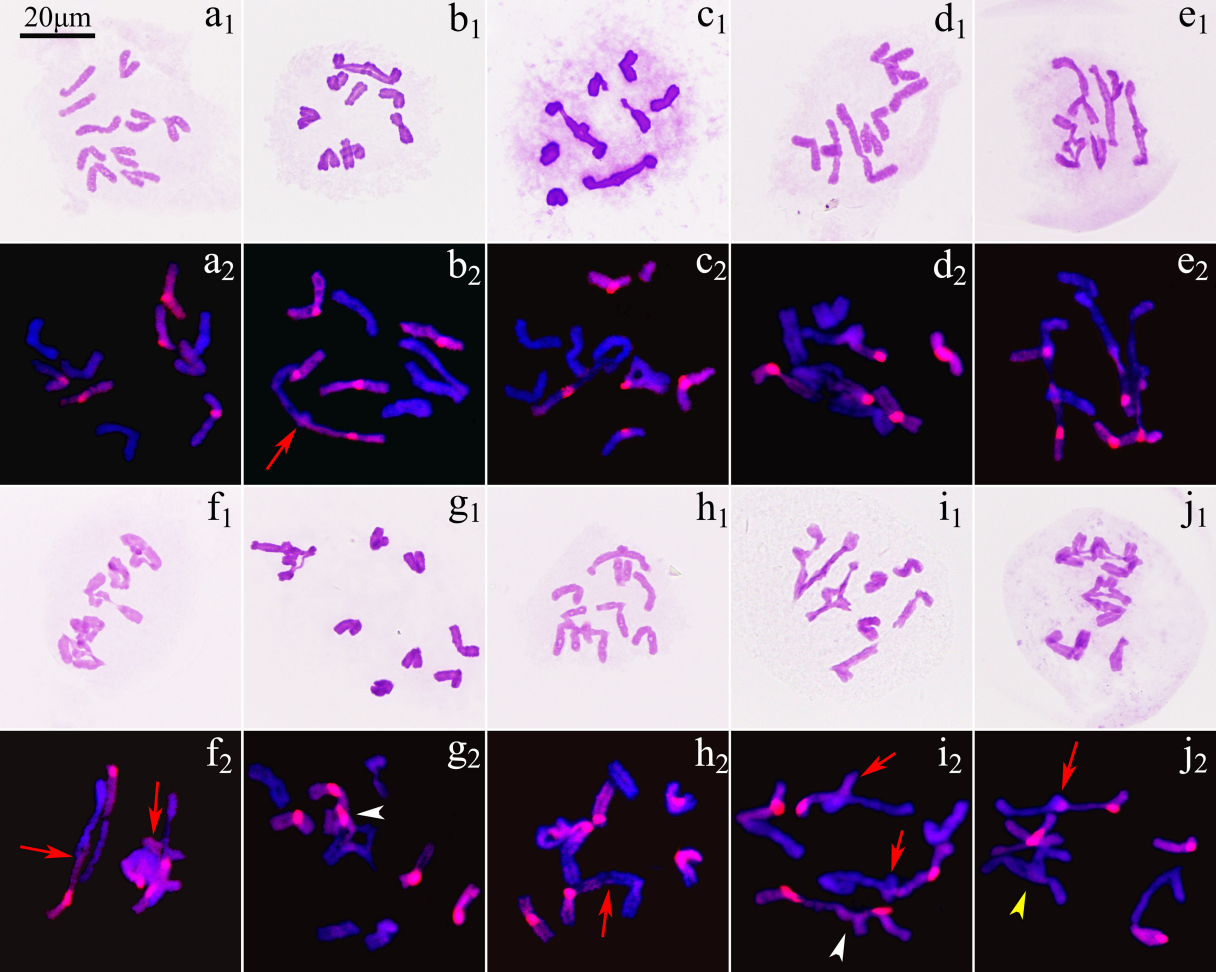
Chromosome pairing in metaphase I of ‘Golden Isles’ (*2n*=2*x*=10, AB). **(a)** absence of pairing; **(b)** 1II^AB^+8I; **(c)** 2II^AB^+6I; **(d)** 3II^AB^+4I; **(e)** 4II^AB^+2I; **(f)** 5II^AB^; **(g)** 1III^ABB^+7I; **(h)** 1III^AAB^+1II^AB^+5I; **(i)** 1III^BAB^+2II^AB^+3I; **(j)** 1III^ABB^+2II^AB^+1II^AA^+1I (red arrow: inter-genome pairing and translocation, yellow arrow head: pairing in A-genome, white arrow head: pairing in B-genome).

**Figure 4:**
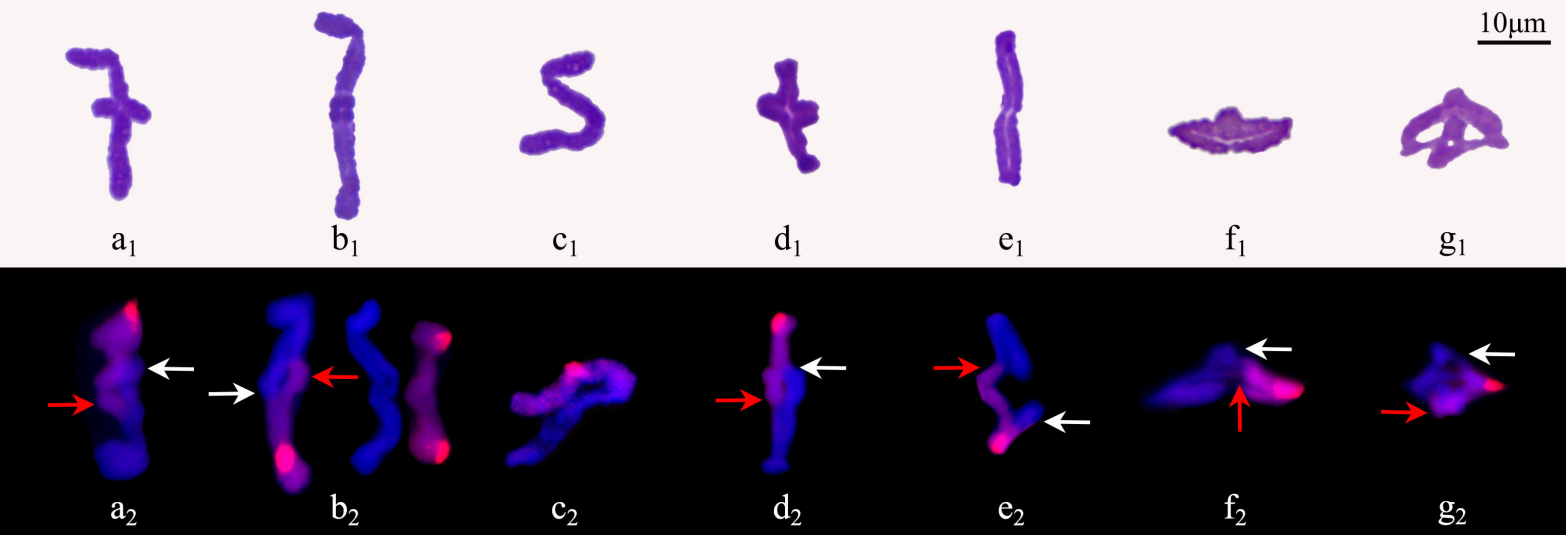
Morphology and constitute of bivalents in metaphase I of ‘Golden Isles’ (2*n*=2*x*=10, AB). **(a–e)**: rod bivalents with one chiasma on each, **(f, g)**: ring bivalents with two chiasmata on each; 1: bivalents stained with Carbol-Fuchsin, 2: the chromosomes from B-genome (red) were identified by GISH; inter-genome translocated segments were indicated by arrows (white for A-genome, and red for B-genome).

**Figure 5:**
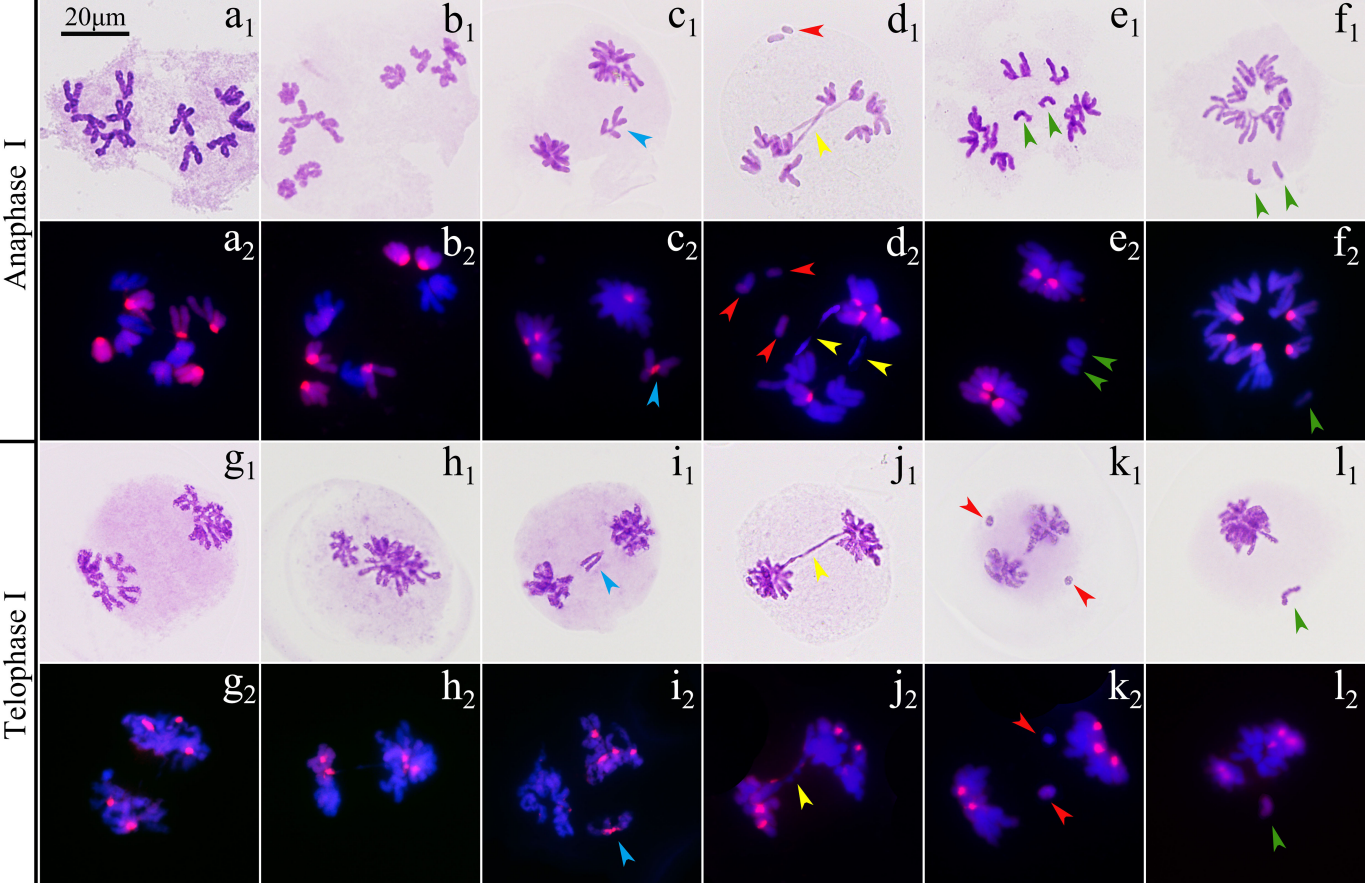
Chromosome behaviors in anaphase I and telophase I of ‘Golden Isles’ (2*n*=2*x*=10, AB). **(a–f)**: anaphase I, **(g–l)**: telophase I; **(a, g)**: equational separation, **(b, h)**: unequal separation, **(c, i)**: lagging chromosome, **(d, j, k)**: chromosome bridge and fragments, e: premature separation of sister chromatids, **(f, l)**: chromosomes failed in separation; 1: stained with Carbol-Fuchsin solution; 2: the chromosomes from A-genome (blue) were stained with DAPI, and those from B-genome (red) were identified by GISH; blue arrow head: lagging chromosome, yellow arrow head: chromosome bridge, red arrow head: fragment, green arrow head: premature separated sister chromatid.

The observation on 471 pollen mother cells (PMCs) of ‘GI’ in metaphase I by Carbol-Fuchsin staining indicated that the mean meiosis configuration was 2*n*=5.8I+1.84II+0.04III+0.01IV, the pairing index was 15.79%, and about 11.06% of the PMCs showed no chromosome pairing (**Figure 3**). The PMCs bearing polyvalents accounted for about 9.98% of the total number. Monovalents were found in 98.09% of the PMCs (**Supplementary Table S4**). GISH was used to observe the 198 PMCs in metaphase I. The results showed that the proportion of three types of bivalents (**Figure 3**), II^AB^, II^AA^ and II^BB^, were 92.83%, 1.79% and 5.38%, respectively. Inter-genome translocations were observed in II^AB^ bivalents. The proportion of rod bivalents with single chiasma was 89.00%, which was much higher than the ring bivalents with double chiasmata, indicating that the probability of simultaneous translocations in both long and short arms was much lower than that of single translocation in only one arm (**Figure 4**). The proportion of abnormal PMCs in anaphase I was about 75.82%, and the abnormal chromosome behaviors included unequal segregation, lagging chromosomes, chromosome bridges and fragments, and premature separation of sister chromatids (**Figure 5**). In 15.38% of the PMCs, the chromosomes did not migrate to the poles, resulted in monocytes in telophase I, indicating that a considerable proportion of PMCs did not form reduced nuclei at the end of the meiotic first division.

**Figure 6:**
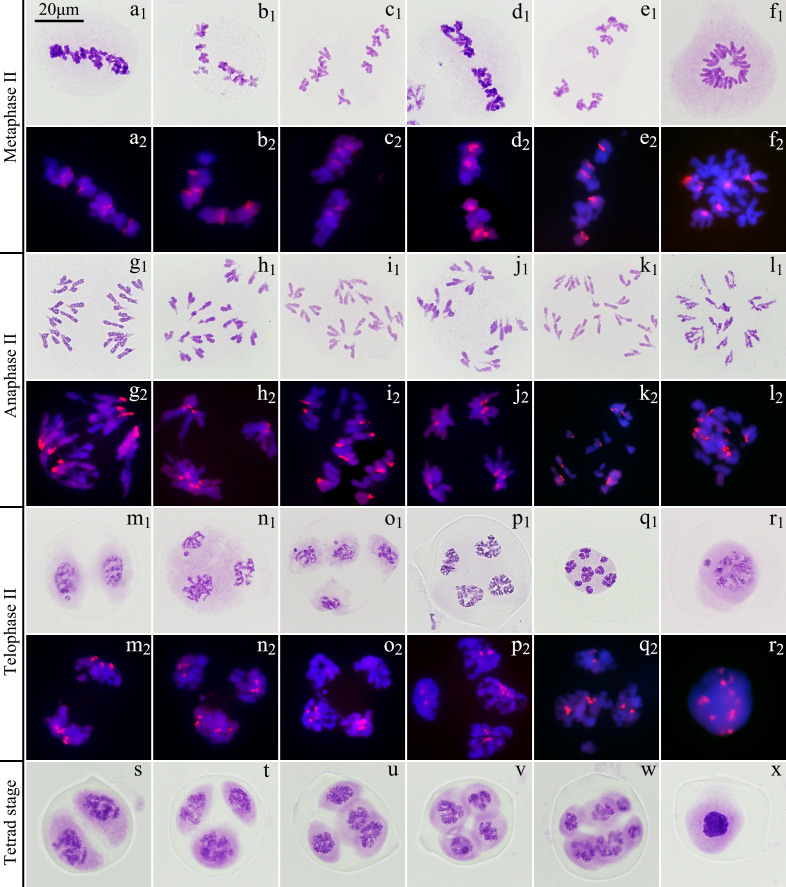
Chromosome behaviors in metaphase II to tetrad stage in ‘Golden Isles’ (2*n*=2*x*=10, AB). **(a)**: fused spindles, **(b)**: tripolar spindles, **(c)**: parallel spindles, **(d)**: linear spindles, **(e)**: multipolar spindles, **(f)**: failure to form spindles; **(g–l)**: separation of sister chromatids in telophase II from different types of spindles; **(m–r)**: different number of nuclei resulted from different types of spindles; **(s)**: dyad, **(t)**: triad, **(u, v)**: tetrad, **(w)**: polyad, **(x)**: monad; 1: stained with Carbol-Fuchsin solution, 2: the chromosomes from A-genome (blue) were stained with DAPI, and those from B-genome (red) were identified by GISH.

Among the 655 PMCs in tetrad phase, the proportion of monads, dyads and triads was 1.83%, 29.77% and 12.98%, respectively (**Figure 6**; **Supplementary Table S4**), while the tetrads without extra microspores or micronuclei accounted for 22.90%, which meant that the proportion of unreduced gametes was up to 37.79% ((2×29.77 + 12.98)/(1.83 + 2×29.77 + 3×12.98 + 4×22.90) × 100%). In the various spindle orientations of metaphase II, fused spindles accounted for 32.06% and tripolar spindles accounted for 15.68%, indicating that most of the unreduced gametes produced by ‘GI’ might be caused by these two spindle orientation anomalies, and belonging to the first division restitution (FDR) type. Since about 15.38% of the PMCs in the first division of meiosis did not form reduced nuclei, these PMCs would form fused spindles and dyads in the second division of meiosis. Therefore, the unreduced gametes formed through this pathway account for about 16.03% of the total gametes and 42.42% of all unreduced gametes.

## 4 Discussion

### 4.1 The origin of allotetraploid tree peony

Karyotype analysis based on GISH and FISH showed that 10 of the 20 chromosomes of ‘GE’ (2*n*=4*x*=20, AABB) were probably derived from the FDR type unreduced gamete produced by its diploid parent ‘GI’ (2*n*=2*x*=10, AB), in which the reciprocal inter-genome translocations were speculated to take place between 4A and 4B chromosomes in their long arms. Meiosis studies verified that ‘GI’ can indeed produce a high proportion (37.97%) of unreduced gametes. These results indicate that 2*n* gametes produced by diploid inter-subsectional hybrid tree peony (*P. × lemoinei*) played an important role in the origination of allotetraploid tree peony, similar to that of the allotriploid tree peony (Zhong et al., 2024a). The rest 10 chromosomes of ‘GE’ also contain both A and B genomes, with inter-genome translocations in 2B and 3B chromosomes, implying that the unknown pollen parent of ‘GE’ is also *P. × lemoinei*.

Meiosis studies have shown that the diploids of *P. × lemoinei* are prone to gengerate translocations in the long arms, and occasionally in both the long and short arms (**Figure 4**, unpublished data). Therefore, the pollen parent of ‘GE’ might also be another diploid of *P. × lemoinei*. However, the fertile 2*n* (2*x*) gametes from diploid hybrids are rare in most cases, as a result, the probability of producing tetraploids by the cross of female and male 2*n* gametes is extremely low (De Storme and Mason, 2014; Ramsey and Schemske, 1998). In contrast, allotetraploids are more fertile and easier to produce 2*x* gametes (Alix et al., 2017). The earliest fertile individuals in *P. × lemoinei* can be traced back to the first two F_2_ plants produced before 1950s, followed by a number of fertile ones obtained in advanced generation hybridizations (Page, 2005; Wister, 1995). The fertility restoration of hybrids is often accompanied by ploidy increasement (Abbott et al., 2013; Alix et al., 2017). Hence, it is speculated that most of these fertile offsprings might be tetraploids (Zhong et al., 2023). So, it is more likely that the unknown pollen parent of ‘GE’ was another allotetraploid tree peony produced earlier than ‘GE’, which was generated in 1980s.

Taken together, ‘GE’ might not be the first allotetraploid tree peony, but they may share the similar genome constitutes. The earliest tetraploid tree peonies are probably those two F_2_ plants, which might be produced by the hybridization of female and male 2*n* gametes produced by F_1_ diploids in *P. × lemoinei*. The earliest formation of allotetraploid tree peonies is a minimal probability event, which indeed took place only a few decades ago. Once the first allotetraploids are formed, they can hybridize with each other or with diploid F_1_ hybrids, and it is possible to produce more allotetraploids (Ramsey and Schemske, 1998).

### 4.2 Inter-subsectional allotetraploid formation vs. intra-subsectional homoploid hybrid speciation in section *Moutan*

Hybridization is not only regarded as evolutionary noise, but also recognized as a creative evolutionary force in plant speciation and evolution (Mallet, 2007; Soltis and Soltis, 2009). Compared with the frequent allopolyploid speciation, homoploid hybrid speciation is much less common (Long and Rieseberg, 2024). However, in *Paeonia* section *Moutan*, allotetraploid formation has only taken place in inter-subsectional hybridization, between *P*. *delavayi* from subsection *Delavayanae* and *P. suffruticosa* from subsection *Vaginatae* (Zhong et al., 2024b, 2023). While the hybridization among close relative species within subsection *Vaginatae* always produce homoploid hybrids or even hybrid species. Homoploid hybridization involving five wild species in subsection *Vaginatae* that people transplanted to their gardens could have given rise to the diverse cultivars of *P. suffruticosa*, the cultivated tree peony, which are now grown all over the world (Zhou et al., 2014). In addition, *P.* × *yananensis* is documented as a naturally formed homoploid hybrid species, with *P. jishanensis* as the maternal parent and *P. rockii* as the paternal parent (Yuan et al., 2010).

Unreduced (2*n*) gametes are crucial for sexual polyploidization in plants (De Storme and Geelen, 2013). The probability of 2*n* gamete production is much higher in hybrid plants than in non-hybrid ones (Ramsey and Schemske, 1998). All the species in section *Moutan* is very stable in ploidy (diploid, 2*n*=2*x*=10) (Hong, 2021), suggesting that the extremely rare natural production of 2*n* gametes might be common in plants of section *Moutan*. Due to the triploid block, it is difficult to obtain triploid hybrid offsprings by crossing 2*n* gametes with normal gametes, but easier to obtain tetraploids by crossing between 2*n* gametes (Ramsey and Schemske, 1998). But when the female 2*n* gametes artificially induced in *P. ostii* by high temperature treatment were open pollinated, only a small number of triploid plants, but no tetraploid ones, were obtained (Liu et al., 2023), indicating that it is definitely difficult for tree peonies to produce 2*n* gametes naturally. Nevertheless, 2*n* gamete generation have been observable in inter-subsectional hybrid tree peonies (Zhong et al., 2024a, 2019), sometimes in a high frequency (this research).

The genomic differentiation between progenitor taxa influences the likelihood of diploid (homoploid) versus polyploid hybrid speciation, because genetic divergence between parents of allopolyploids is found to be significantly greater than in the case of homoploid hybrid species (Paun et al., 2009). Only when there is a suitable (neither too low nor too high) level of divergence between the parental taxa, they can produce the diploid F_1_ hybrids which are capable to produce 2*n* gametes in a high frequency (Sang et al., 2004). Most F_1_ hybrids observed to produce polyploids were highly sterile (Ramsey and Schemske, 1998). The crossing between inter- or intra-subsectional species of section *Moutan* both generates homoploid hybrids in F_1_ generation, but with diverse difficulty. The intra-subsectional hybridization is easy to get viable seeds and fertile hybrids, while the inter-subsectional hybridization only generates few viable seeds and fewer sterile hybrids (Wang et al., 2013), which implying that severe incompatibility exists between inter-subsectional species, instead of intra-subsectional species.

Therefore, the suitable level of divergence between the parents of inter-subsectional hybridization, *P. delavayi* and *P. suffruticosa*, which belong to subsection *Delavayanae* and subsection *Vaginatae*, respectively, probably promotes the polyploidization of tree peonies by facilitating 2*n* gamete production.

### 4.3 Comparison of polyploidization mechanisms between section *Moutan* and section *Paeonia*: similarities and differences

Different from the status in section *Moutan*, polyploid speciation is quite common in section *Paeonia*. In the 22 tetraploid taxa, including 8 species and 14 subspecies, of section *Paeonia*, only four taxa are autotetraploids, whereas 18 are allotetraploids. Therein, 8 allotetraploid taxa are formed by hybridization and polyploidization between diploid taxa, while 10 are derived from homoploid tetraploid hybridization (Zhou et al., 2021), implying that the hybridization among diploid taxa play an important role in polyploidization in section *Paeonia*. Unlike the different level of compatibility between inter- and intra-subsectional hybridization in section *Moutan*, almost all the diploid species in section *Paeonia* are intersterile, including those involved in hybridization and polyploidization (Hong, 2021; Saunders and Stebbins, 1938; Zhou et al., 2021). Hence, the mechanism of allotetraploidization in section *Paeonia* is consistent with that in section *Moutan*, in which the key point is the hybridization between divergent diploid parents with significant incompatibility. Additionally, there are totally 5 pairs of parents involving 6 diploid taxa, which contribute to the hybridization and polyploidization in section *Paeonia* (Zhou et al., 2021). The parents of each pair uniformly diverged 18.72~21.61 Mya, approximate with 22.40 Mya, the time of divergence between *P. delavayi* (subsection *Delavayanae*) and *P. suffruticosa* (subsection *Vaginatae*) in section *Moutan* (**Table 1**). Therefore, the divergence level (18.72~ 22.40 Mya) of diploid parents might be the universally fundamental element in hybridization and polyploidization in genus *Paeonia*.

**Table 1:** Divergence time of the diploid parents involved in hybridization and polyploidization in section *Paeonia* (Zhou et al., 2021).

Tetraploid taxon	Maternal parent	Paternal parent	Divergence time of parents (Mya)
*Paeonia. coriacea*	*P. algeriensis*	*P. obovata*	18.72
*P. kesrouanensis*	*P. corsica*	*P. obovata*	18.72
*P. mairei*	*P. veitchii*	*P. obovata*	21.61
*P. officinalis* subsp. *banatica*	*P. obovata*	*P. tenuifolia*	21.61
*P. officinalis* subsp. *huthii*	*P. obovata*	*P. tenuifolia*	21.61
*P. officinalis* subsp. *microcarpa*	*P. obovata*	*P. tenuifolia*	21.61
*P. officinalis* subsp. *officinalis*	*P. obovata*	*P. tenuifolia*	21.61
*P. peregrina*	*P. daurica*	*P. tenuifolia*	21.61

The main difference is that allotetraploidization occurred in nature in section *Paeonia*, but in gardens in section *Moutan*. The habitats of species within subsection *Vaginatae* are completely isolated from those of subsection *Delavayanae* by the severe ecological shifts of the Hengduan Mountains, Southwestern China (Hong, 2010; Zhou et al., 2014), which might eliminate the possibility of their natural hybridization. In contrast, the diploid parents of allotetraploids in section *Paeonia* are sympatric (e.g. *P. veitchii* vs. *P. obovata*, and *P. daurica* vs. *P. tenuifolia*) (Hong, 2010), or might be once sympatric (e.g. *P. obovata* vs. other species) (Zhou et al., 2021), with opportunities to hybridize in nature. Hence, the allotetraploidization in section *Moutan* shares the same mechanism with that in section *Paeonia*, but in a different way to make it realized, that is with the assist from mankind or not.

Furthermore, four autotetraploid taxa are found in section *Paeonia*, while none in section *Moutan* (Zhou et al., 2021), suggesting that the probability of 2*n* gametes production in non-hybrid plants of the two sections might be very different. Some tetraploid plants were obtained from the cross between induced 2*n* gametes and natural gametes from *P*. *lactiflora*, a diploid species of section *Paeonia* (Zhu et al., 2022), indicating that *P*. *lactiflora* can produce some 2*n* gametes naturally. However, the induced 2*n* gametes of *P*. *ostii*, a diploid species of section *Moutan*, could only obtain triploids, but not tetraploids, through open pollination (Liu et al., 2023), indicating that *P*. *ostii* can hardly produce 2*n* gametes naturally. This difference might be the main reason for the inability of section *Moutan* to produce autotetraploids naturally.

### 4.4 Life history divergence is likely to result in the different patterns of polyploidization, as well as speciation and evolution in genus *Paeonia*

**Figure 7:**
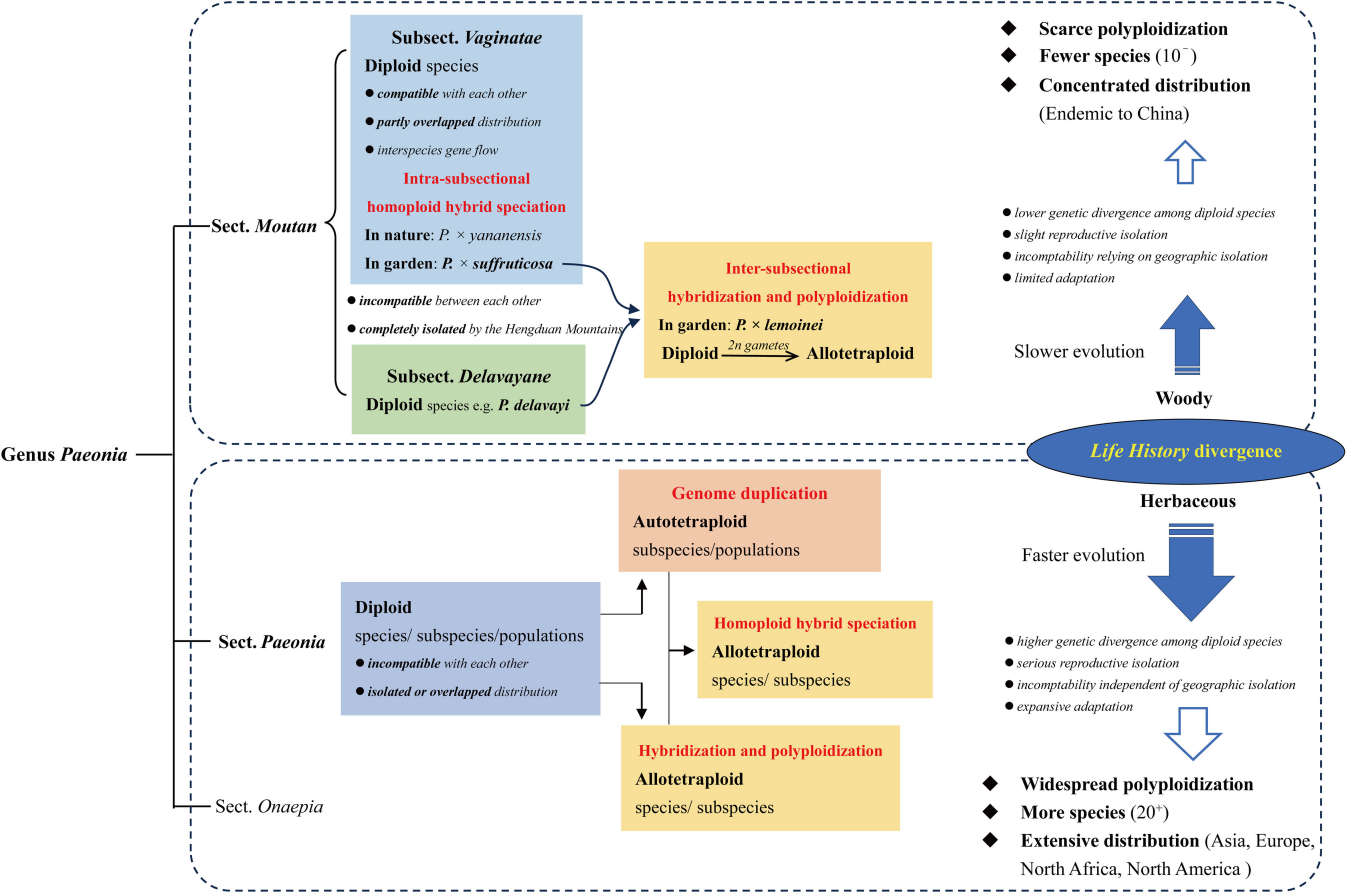
Summary of polyploidization mechanisms in genus *Paeonia.* The different life history between section *Moutan* and section *Paeonia*, which is woody and herbaceous, respectively, could be the main contributor to their diverse level of polyploidization, and different tempo of speciation and evolution.

As summarized above, interparent incompatibility is necessary for allopolyploidization in genus *Paeonia*. However, intersterility exists not only between species from different clades that diverged earlier than 18.72 Mya both in section *Moutan* and section *Paeonia*, but also exits between those from the clades that diverged 9.34~14.08 Mya in section *Paeonia* (Zhou et al., 2021). While in section *Moutan*, *P. rockii* is compatible with other species, including *P. jishanensis* and *P. ostii*, that diverged 12.06 Mya from it (Zhou et al., 2021), implying that the level of incompatibility is in a different proportion with genetic divergence in section *Paeonia*, from that in section *Moutan*. Moreover, there are much more clades and species (over twice as much), and much wider distribution ranges, in section *Paeonia*, than in section *Moutan*, indicating the different tempo of speciation and habitat expansion between them (**Figure 7**).

One of the most divergent traits between section *Moutan* and section *Paeonia* is life history, which is woody and herbaceous, respectively. Rates of molecular evolution are linked to life history in flowering plants, which are consistently low in trees and shrubs, as compared with related herbaceous plants (Gaut et al., 2011; Smith and Donoghue, 2008). The positive relationship between species numbers and the rate of neutral molecular evolution have been demonstrated in flowering plants (Barraclough and Savolainen, 2001). Reproductive isolation is positively correlated with genetic distance, and the postzygotic incompatibilities could accumulate and even increase faster than a linear rate with time (Eric et al., 2015). In turn, reproductive isolation can also facilitate the accumulation of genetic differences, with the resulting feedback loop, given enough time, usually leads to complete genetic isolation (Bock et al., 2023; Rieseberg and Willis, 2007). The establishment of reproductive barriers is not only the prerequisite for species formation (Fernández-Mazuecos and Glover, 2017), but also important for allopolyploidization (Ramsey and Schemske, 1998).

In addition, the rate of climatic niche evolution is also growth-form-dependent, namely, woody lineages accumulate fewer changes per million years in climatic niche space, and explore smaller climate space than related herbaceous lineages (Smith and Beaulieu, 2009). Adaptive evolution in plants is largely connected with genomic variations, which could affect genome size variation, gene expression, phenotype variation, and adaptation (Hu et al., 2023). Moreover, ecological adaptation is the major driver of reproductive isolation (Sobel et al., 2010). The Pan-Himalaya is deemed to be the refugium of both woody and herbaceous peonies. There were five vicariance events and 21 dispersal events in the evolutionary history of genus *Paeonia* (Zhou et al., 2021). Only one vicariance event and five dispersal events are involved in the speciation and evolution of woody peonies. The woody and herbaceous peonies both dispersed into the adjacent areas, including East Asia, in the early dispersal events. But only the herbaceous ones dispersed latter to Central and West Asia, North America, Europe, and North Africa (Hong, 2010; Zhou et al., 2021), where the climatic oscillations and sea level eustacy might have caused isolation and secondary contacts of the previously isolated species, giving the opportunity of hybridization (Mao et al., 2021; Zhou et al., 2021). Repeated cycles of connectivity and isolation may act as drivers of species diversification (Mosbrugger et al., 2018), as well as allopolyploidization (Stebbins, 1985).

Therefore, the different life history between section *Moutan* and section *Paeonia*, which is woody and herbaceous, respectively, could be the main contributor to their diverse patterns of polyploidization, and even speciation and evolution (**Figure 7**).

## 5 Conclusion

The distant hybridization between intersterile species from different subsections of section *Moutan* probably promotes the tetraploidization of tree peonies by facilitating 2*n* gamete production. The mechanism of tetraploidization in section *Moutan* is highly consistent with that in section *Paeonia*, but needs the assist from mankind. The divergence of life history between tree peonies and herbaceous peonies is speculated to contribute to the different level of polyploidization, and the distinct tempo of speciation and evolution, between section *Moutan* and section *Paeonia* in genus *Paeonia*.

## Data Availability

The original contributions presented in the study are included in the article/**Supplementary Material**. Further inquiries can be directed to the corresponding authors.
