# Supplementary material for: The mechanism of tetraploidization in tree peony, and its implications for speciation and evolution of genus Paeonia L
Source: Front Plant Sci. 2025 May 12;16:1586225. doi: 10.3389/fpls.2025.1586225 (PMC12104305; doi:10.3389/fpls.2025.1586225)
Supplement: Supplementary file 1 [file DataSheet1.zip › Supplementary files/Table S1 Karyotype data of ‘Golden Era’ (2n=4x=20, AABB).docx]

**Table S1 Karyotype data of ‘Golden Era’ (2*n*=4*x*=20, AABB)**

| **Chromosome No.** | **Relative length (%, mean** ± **SD)** | **Arm ratio (mean** ± **SD)** | **Chromosome type** | **45S rDNA** | **5S rDNA** | **Relative length of inter-genome translocation (%, mean ± SD)** |
| --- | --- | --- | --- | --- | --- | --- |
| 1^1^A | 5.88±0.28 | 1.43±0.24 | m |  |  |  |
| 1^2^A | 5.55±0.31 | 1.32±0.15 | m |  |  |  |
| 2^1^A | 5.45±0.17 | 1.21±0.18 | m |  |  |  |
| 2^2^A | 5.17±0.18 | 1.21±0.19 | m |  |  |  |
| 3^1^A | 5.64±0.26 | 1.12±0.05 | m | + | ++ |  |
| 3^2^A | 5.44±0.21 | 1.28±0.15 | m | + | ++ |  |
| 4^1^A | 5.11±0.26 | 1.52±0.10 | m | + |  |  |
| 4^2^A | 4.86±0.24 | 1.53±0.11 | m | + |  | 2.15±0.20 (long arm) |
| 5^1^A | 4.44±0.23 | 3.48±0.68 | st | + |  |  |
| 5^2^A | 4.25±0.33 | 3.15±0.39 | st | + |  |  |
| 1^1^B | 5.25±0.21 | 1.37±0.12 | m |  |  |  |
| 1^2^B | 4.96±0.24 | 1.45±0.16 | m |  |  |  |
| 2^1^B | 5.31±0.38 | 1.20±0.21 | m | + |  | 1.80±0.25 (long arm) |
| 2^2^B | 5.04±0.23 | 1.14±0.15 | m | + |  |  |
| 3^1^B | 5.05±0.23 | 1.17±0.08 | m | + | ++ |  |
| 3^2^B | 4.98±0.21 | 1.12±0.12 | m | + | ++ | 0.56±0.08 (short arm)  0.41±0.16 (long arm) |
| 4^1^B | 4.72±0.20 | 1.71±0.27 | sm | + |  | 2.14±0.28 (long arm) |
| 4^2^B | 4.65±0.17 | 1.50±0.22 | m | + |  |  |
| 5^1^B | 4.15±0.36 | 3.26±0.28 | st | + |  |  |
| 5^2^B | 4.09±0.26 | 2.84±0.33 | sm | + |  |  |
