# Supplementary material for: The mechanism of tetraploidization in tree peony, and its implications for speciation and evolution of genus Paeonia L
Source: Front Plant Sci. 2025 May 12;16:1586225. doi: 10.3389/fpls.2025.1586225 (PMC12104305; doi:10.3389/fpls.2025.1586225)
Supplement: Supplementary file 1 [file DataSheet1.zip › Supplementary files/Table S2 Karyotype data of ‘Golden Isles’ (2n=2x=10, AB).docx]

**Table S2 Karyotype data of ‘Golden Isles’ (2*n*=2*x*=10, AB)**

| **Chromosome No.** | **Relative length**  **(%, mean** ± **SD)** | **Arm ratio**  **(mean** ± **SD)** | **Chromosome type** | **45S rDNA** | **5S rDNA** |
| --- | --- | --- | --- | --- | --- |
| 1A | 11.34±0.51 | 1.34±0.25 | m |  |  |
| 2A | 10.55±0.76 | 1.15±0.15 | m |  |  |
| 3A | 11.28±0.60 | 1.17±0.12 | m | + | ++ |
| 4A | 10.27±0.53 | 1.59±0.12 | m | + |  |
| 5A | 8.29±0.45 | 3.33±0.37 | st | + |  |
| 1B | 10.75±0.85 | 1.4±0.25 | m |  |  |
| 2B | 10.25±0.64 | 1.16±0.22 | m | + |  |
| 3B | 9.79±0.41 | 1.17±0.11 | m | + | ++ |
| 4B | 9.14±0.30 | 1.54±0.28 | m | + |  |
| 5B | 8.35±0.49 | 2.97±0.32 | sm | + |  |
