# Supplementary material for: The mechanism of tetraploidization in tree peony, and its implications for speciation and evolution of genus Paeonia L
Source: Front Plant Sci. 2025 May 12;16:1586225. doi: 10.3389/fpls.2025.1586225 (PMC12104305; doi:10.3389/fpls.2025.1586225)
Supplement: Supplementary file 1 [file DataSheet1.zip › Supplementary files/Table S3 Comparison on arm ratios of homologous chromosomes between ‘Golden Isles’ and ‘Golden Era’ (p-value).docx]

**Table S3 Comparison on arm ratios of homologous chromosomes between ‘Golden Isles’ and ‘Golden Era’ (*p*-value)**

| **Chromosome No. of ‘Golden Era’** | **Chromosome No. of ‘Golden Isles’** | | | | | | | | | |
| --- | --- | --- | --- | --- | --- | --- | --- | --- | --- | --- |
|  | **1A** | **2A** | **3A** | **4A** | **5A** | **1B** | **2B** | **3B** | **4B** | **5B** |
| **1^1^A** | 0.503 |  |  |  |  |  |  |  |  |  |
| **1^2^A** | 0.883 |  |  |  |  |  |  |  |  |  |
| **2^1^A** |  | 0.635 |  |  |  |  |  |  |  |  |
| **2^2^A** |  | 0.658 |  |  |  |  |  |  |  |  |
| **3^1^A** |  |  | 0.747 |  |  |  |  |  |  |  |
| **3^2^A** |  |  | 0.384 |  |  |  |  |  |  |  |
| **4^1^A** |  |  |  | 0.589 |  |  |  |  |  |  |
| **4^2^A** |  |  |  | 0.645 |  |  |  |  |  |  |
| **5^1^A** |  |  |  |  | 0.261 |  |  |  |  |  |
| **5^2^A** |  |  |  |  | 0.198 |  |  |  |  |  |
| **1^1^B** |  |  |  |  |  | 0.753 |  |  |  |  |
| **1^2^B** |  |  |  |  |  | 0.633 |  |  |  |  |
| **2^1^B** |  |  |  |  |  |  | 0.670 |  |  |  |
| **2^2^B** |  |  |  |  |  |  | 0.815 |  |  |  |
| **3^1^B** |  |  |  |  |  |  |  | 0.980 |  |  |
| **3^2^B** |  |  |  |  |  |  |  | 0.621 |  |  |
| **4^1^B** |  |  |  |  |  |  |  |  | 0.098 |  |
| **4^2^B** |  |  |  |  |  |  |  |  | 0.704 |  |
| **5^1^B** |  |  |  |  |  |  |  |  |  | **0.008** |
| **5^2^B** |  |  |  |  |  |  |  |  |  | 0.190 |
