# Supplementary material for: The mechanism of tetraploidization in tree peony, and its implications for speciation and evolution of genus Paeonia L
Source: Front Plant Sci. 2025 May 12;16:1586225. doi: 10.3389/fpls.2025.1586225 (PMC12104305; doi:10.3389/fpls.2025.1586225)
Supplement: Supplementary file 1 [file DataSheet1.zip › Supplementary files/Table S4 Meiotic abnormalities observed in ‘Golden Isles’ (2n=2x=10, AB).docx]

**Table S3 Meiotic abnormalities observed in ‘Golden Isles’ (2*n*=2*x*=10, AB)**

| **Phases** | **No. of analyzed cells** | **Percentage of abnormal cells (%)** | **Abnormalities** | **No. of cells** | **Percentage (%)** |
| --- | --- | --- | --- | --- | --- |
| Metaphase I | 471 | 98.09 | Univalents | 462 | 98.09 |
|  |  |  | Polyvalents | 47 | 9.98 |
| Anaphase I | 273 | 75.82 | Lagging chromosomes | 68 | 24.91 |
|  |  |  | Bridge and/or fragments | 38 | 13.92 |
|  |  |  | Non-separation | 42 | 15.38 |
|  |  |  | Unequal separation | 59 | 21.61 |
| Telophase I | 59 | 57.63 | Bridge | 13 | 22.03 |
|  |  |  | Micronuclei | 21 | 35.59 |
| Metaphase II | 287 | 79.79 | Fused spindles | 92 | 32.06 |
|  |  |  | Multiple spindles | 52 | 18.12 |
|  |  |  | Parallel spindles | 13 | 4.53 |
|  |  |  | Tripolar spindles | 45 | 15.68 |
|  |  |  | Bridge and/or fragments | 14 | 4.88 |
|  |  |  | Free chromosome | 13 | 4.53 |
| Anaphase II | 395 | 84.81 | One group (of chromosomes) | 1 | 0.25 |
|  |  |  | Two groups | 111 | 28.10 |
|  |  |  | Three groups | 66 | 16.71 |
|  |  |  | Five or more groups | 55 | 13.92 |
|  |  |  | Lagging chromosomes | 46 | 11.65 |
|  |  |  | Bridge and/or fragments | 56 | 14.18 |
| Telophase II | 368 | 79.62 | One nucleus | 4 | 1.09 |
|  |  |  | Two nuclei | 79 | 21.47 |
|  |  |  | Three nuclei | 29 | 7.88 |
|  |  |  | Five or more nuclei | 53 | 14.40 |
|  |  |  | Micronuclei | 128 | 34.78 |
| Tetrad phase | 655 | 77.10 | Monad | 12 | 1.83 |
|  |  |  | Dyad | 195 | 29.77 |
|  |  |  | Triad | 85 | 12.98 |
|  |  |  | Polyad | 34 | 5.19 |
|  |  |  | Tetrad with extra microspores or micronuclei | 179 | 27.33 |
